# 

**Published:** 1826-10

**Authors:** 


### MONTHLY LIST OF MEDICAL BOOKS.

[*No books can be entered on this List except those sent to us for the purpose; as, in the list hitherto transmitted, the names of works have frequently been given as published, which have not appeared for weeks, or even months, after.*]

A Lecture on the Uses of Anatomy and Physiology in various Branches of Knowledge, delivered on the 1st of November, 1824, by James Macartney, M.D. F.R.S. F.L.S. M.R.I.A. Professor of Anatomy and Surgery in the University of Dublin, &c.—Dublin, 1826.

Callow and Wilson's Catalogue of Modern Medical Books, for 1826.

A Catalogue of Second-hand and Scarce Medical Books, on Sale by Callow and Wilson, No. 16, Princes-street, Soho, London. (No. 2.)

A Comparative View of the more intimate Nature of Fever: deduced from Physiological Analysis, and illustrated by Critical Remarks and Practical Observations. By JAMES BLACK, M.D. S.R.N. &c.—London, 1826.

Part Third of a Series of Elementary Lectures on the Veterinary Art: wherein the Anatomy, Physiology, and Pathology of the Horse, are essayed on the general Principles of Medical Science. By WILLIAM PERCIVAL, Member of the Royal College of Surgeons, &c.—London, 1826.

Observations on the Medical Character. Addressed to the Graduates of the College of Physicians and Surgeons of New York, at the Commencement, held on the 4th of April, 1826. By DAVID HOSACK, M.D. &c.—New York, 1826.

An Introductory Lecture delivered in the College of Physicians and Surgeons, at the Opening of the Winter Session, on the 7th of November, 1825. By DAVID HOSACK, M.D. &c. —New York, 1825.

### METEOROLOGICAL JOURNAL,

### NOTICE TO CORRESPONDENTS.

*Communications have been received from* Dr. Gregory, Mr. Stone, Dr. Hawkins, Mr. Boyle, Mr. Sym, *and* " A Physician."

